# Optimisation of metabolic dysfunction-associated steatotic liver disease (MASLD) screening algorithm for resource-poor settings using machine learning

**DOI:** 10.1371/journal.pone.0352231

**Published:** 2026-09-24

**Authors:** Chamila Mettananda, Kaveesha Sivasumithran, Lakmali Ranaweera, Anjalika Madhubhashini, Chamila Ranawaka, Arunasalam Pathmeswaran, Anuradha Dassanayake

**Affiliations:** 1 Faculty of Medicine, University of Kelaniya, Ragama, Sri Lanka; 2 Faculty of Science, University of Colombo, Colombo, Sri Lanka; 3 North Colombo Teaching Hospital, Ragama, Sri Lanka; Charite Universitatsmedizin Berlin, GERMANY

## Abstract

**Background:**

The European Association for the Study of the Liver (EASL) metabolic dysfunction-associated steatotic liver disease (MASLD) screening algorithm involves two steps: initial screening with FIB-4, followed by referral for vibration-controlled transient elastography (VCTE) in patients likely to have significant fibrosis (SF). However, VCTE is not widely available in resource-limited settings.

**Aim:**

To optimise the EASL MASLD screening algorithm for resource-poor settings using machine learning (ML).

**Methods:**

We analysed data from 964 adults aged ≥35 years who underwent VCTE at a tertiary referral centre in Sri Lanka between November 2024 and 2025. Multiple ML models using different methods and variable combinations were trained on 80% of the dataset and tested on the remaining 20%. The best models were selected based on performance and externally validated on data from 430 patients who underwent VCTE before November 2024. Model performance was compared with that of the FIB-4 score using confusion matrices.

**Results:**

A Random Forest model incorporating age, AST, ALT, and platelet count separately, rather than using the FIB-4 score, outperformed in predicting SF. The model using all variables showed the best predictive performance for SF, with an AUC-ROC of 0.808. The variables used in the model, in descending order of feature importance, were AST, platelet count, BMI, diabetes mellitus, ALT, age, hypertension, dyslipidaemia, sex, family history, diabetes complication, hypothyroidism, and smoking. External validation of the all-variable model demonstrated an AUC of 0.795 and predicted SF in 9.0% more patients without increasing negative VCTE referrals compared with using the FIB-4 score as the screening tool in the first step of the MASLD screening algorithm.

**Conclusions:**

ML-based models were more effective than the FIB-4 score as the first-line screening tool for VCTE referrals, substantially improving the identification of patients with significant fibrosis in this South Asian cohort.

## Introduction

Metabolic dysfunction-associated steatotic liver disease (MASLD) is the most common chronic liver disease in the world, affecting 30–35% [1–4]. It is a spectrum of disease spanning from simple steatosis to cirrhosis [5–7]. Around 10–20% of patients with MASLD progress to advanced chronic liver cell disease (ACLD) and hepatocellular carcinoma over 15–20 years [3,6–10]. Normal liver enzyme levels cannot rule out fibrosis [11,12]. The stage of liver fibrosis predicts disease progression. Patients with significant liver fibrosis (SF) are likely to progress to advanced fibrosis (AF) [13–15]. Therefore, early detection and initiation of liver-directed therapy at the SF stage is recommended [16–19].

The European Society for the Study of Liver (EASL) guideline recommends annual screening of patients with diabetes or MASLD using a two-step process. First, using the FIB-4 score, a non-invasive biochemical test to identify those likely to have SF, and subsequently imaging those likely to have SF using vibration-controlled transient elastography (VCTE) or Magnetic resonance elastography (MRE) as the second step [16,17,20–25].

However, neither VCTE nor MRE is readily available in resource-limited settings; therefore, it is helpful to improve the selection of VCTE further to optimise practical use. The current recommendation in European guidelines is to refer patients with an FIB-4 score ≥1.3 (≥2 in those aged >65 years) for VCTE, but the FIB-4 Score has not been validated in most South Asians [18]. Furthermore, machine learning (ML) models have been shown to perform better at predicting SF than other non-invasive tests, such as FIB-4, FAST, and NFS, across European, American, Chinese, and some Asian populations [26–36].

Therefore, we aimed to develop ML-based models to predict SF using medical history, anthropometric measurements, and freely available blood investigations, and to compare the new models’ predictions with those of the FIB-4 score as the first step in the screening algorithm for MASLD to optimise the utilisation of limited VCTE facilities in resource-limited settings.

## Methods

We used de-identified FibroScan Registry data from patients with VCTE at a tertiary care referral centre in Sri Lanka from 1^st^ November 2024–1^st^ November 2025. This is the only state-sector centre in Sri Lanka with VCTE. We extracted data on 1^st^ November 2025 from patients aged 35 years or older with ultrasonographic (US) evidence of MASLD and complete data to calculate FIB-4, including serum aspartate aminotransferase (AST), alanine aminotransferase (ALT), and platelet count. We excluded patients with US evidence of cirrhosis, and those with MASLD due to causes other than MASLD, such as methotrexate, tamoxifen, and unsafe alcohol intake or hepatitis. We calculated the FIB-4 score of the patients using the following equation [37].

### Model development

We developed several ML models to predict SF using registry data. Participant data were split into two groups: training sample for model development (80%) and testing sample for internal validation (20%). The training sample was used to build the ML models, and the testing sample was used to assess the efficacy of the algorithms trained on it. We used a preprocessing pipeline to eliminate any potential data leakage. The missing data percentage was only 4.5%, so we imputed missing values with train-set-derived imputation parameters using SimpleImputer and subsequently applied them to the test data set. Various combinations of variables were tested to predict SF using variable importance and domain knowledge of SF. We trialled models using the variables in the calculation of FIB-4 (age, platelet count, AST, and ALT values, used individually) and using the calculated FIB-4 score instead. A slight imbalance of the response variable was noted, with 58.51% patients having SF and 41.49% not having SF. To address this, the Synthetic Minority Over-sampling Technique (SMOTE) was used within the modelling pipeline, and models trained on balanced data were compared with those trained on the original data to identify the best model. 5-fold cross-validation on the training set was performed for hyperparameter tuning, using a grid search for each algorithm. Models were trained using different ML algorithms, namely, decision tree, random forest, XGBoost (eXtreme gradient boosting), k-Nearest Neighbours, Support Vector Machine, AdaBoost (adaptive boosting), and Gradient Boosting. Models were built using the publicly available Google Colab ML platform and the Scikit-learn library in Python [9]. The models were compared for predictive performance using accuracy, precision, recall, F1 score, and area under the receiver operating characteristic curve (AUC-ROC). An aggregated ranking for each model was obtained by summing individual metric ranks, and the model with the best ranking was selected as the best model.

We compared the performance of the ML-based model vs the FIB-4 score in predicting SF as the first step in the screening algorithm for MASLD, using the area under the receiver operating characteristic curves (AUC-ROC). Using confusion matrices, we compared the added benefit of the new ML model over the current recommendation for using the FIB-4 score for screening.

### External validation

Next, we externally validated the new ML-based model using a separate, de-identified database of patients with MASLD who had VCTE performed before 1^st^ November 2024. We compared the performance of the new model against the FIB-4 score in predicting SF as the first step in the screening algorithm for MASLD in the external validation cohort, using AUC and confusion matrices.

All experiments were conducted in accordance with the Declaration of Helsinki. Ethical clearance was granted for the FibroScan Registry (P/143/10/2023) and the external validation cohort (P/66/07/2021) by the Ethics Review Committee of the Faculty of Medicine, University of Kelaniya, Sri Lanka. Written informed consent was obtained from all the participants. The authors did not have access to information that could identify individual participants during or after data collection for this secondary analysis.

## Results

ML-based models were developed using the Fatty Liver database. The selected sample consisted of 964 adults: 44% males, mean age of 57.3 years, with 58.5% having SF confirmed on VCTE. Baseline characteristics of the study population are shown in Table 1.

**Table 1:** Baseline characteristics of the cohort used in ML-based model development and validation.

	Total N = 964
Age, mean(SD), years	57.3 (9.6)
Age ≤ 65 years, N (%)	774 (80.3)
Age > 65 years, N (%)	190 (19.7)
Male, N (%)	422 (43.8)
Hypertension, N (%)*	390 (41.1)
Dyslipidaemia, N (%)**	477 (50.4)
Diabetes N (%)	439 (45.5)
Diabetic complications present, N(%)***	38 (5.0)
Hypothyroidism N (%)	78 (11.0)
Current smokers, N (%)	23 (2.8)
Family history of liver disease, N (%)	88 (9.1)
BMI, mean (SD) Kg/m2	25.52 (4.2)
BMI ≥ 23 Kg/m2, N (%)	696 (72.2)

SD standard deviation, N number, BMI Body Mass Index.

Missing values: *16, **17, ***96.

The comparison of the performance metrics of the two models, developed using age, platelet count, AST, and ALT values separately (Model 1) and using the FIB-4 score instead of age, platelet count, AST, and ALT (Model 2), is shown in Table 2. The Model-1, fitted with the Random Forest method, had 77.7% accuracy, 0.771 recall, and 0.808 AUC-ROC, and was selected as the best combination for variable use. Therefore, age, platelet count, AST, and ALT values were used separately in further models.

**Table 2:** Comparison of optimal machine learning models developed using the factors required for the calculation of FIB-4, calculated separately vs as the FIB-4 score in the machine learning model.

ML model	Optimal model	Accuracy	Recall	Precision	F1-Score	AUC-ROC
Model −1	Random Forest on data balanced using SMOTE	77.72%	0.771	0.777	0.773	0.808
Model-2	Random Forest on data balanced using SMOTE	72.02%%	0.723	0.72	0.72	0.776

AUC-ROC: Area Under the Curve – Receiver Operating Curve, ML Machine learning, SMOTE: Synthetic Minority Over-sampling Technique.

Model 1- age, platelet count, AST, and ALT values used separately with other variables.

Model 2 – age, platelet count, AST, and ALT values replaced with the FIB-4 score and used with other variables.

**Fig 1:**
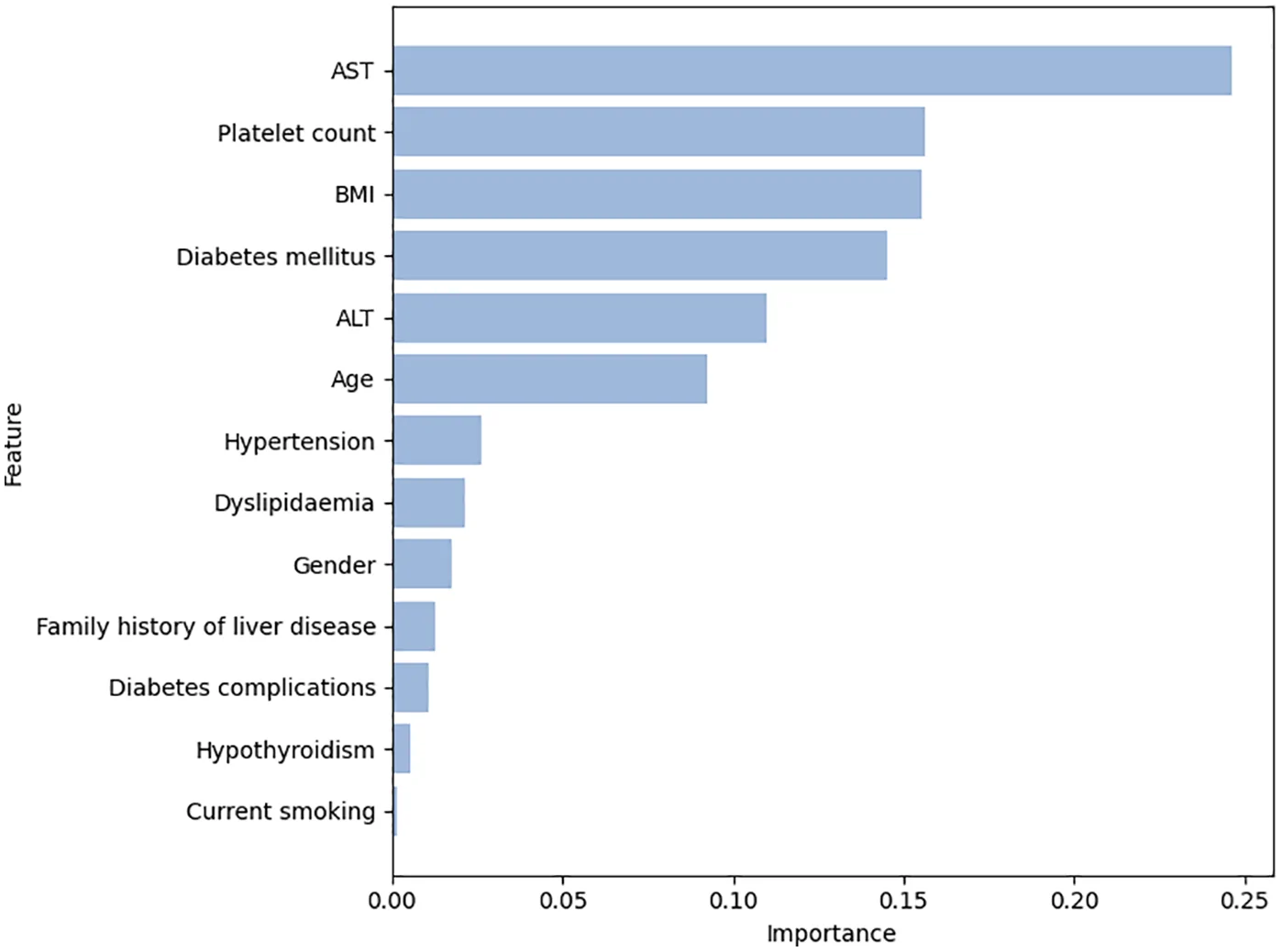
Feature importance of the variables used in the machine learning Model-1. BMI: Body Mass Index, AST: Aspartate aminotransferase, ALT: Alanine aminotransferase.

Feature importances of the variables used in Model 1, in descending order of feature importance, are shown in Fig 1. AST, platelet count, BMI, presence or absence of diabetes mellitus, and ALT were the top five variables associated with the outcome.

**Fig 2:**
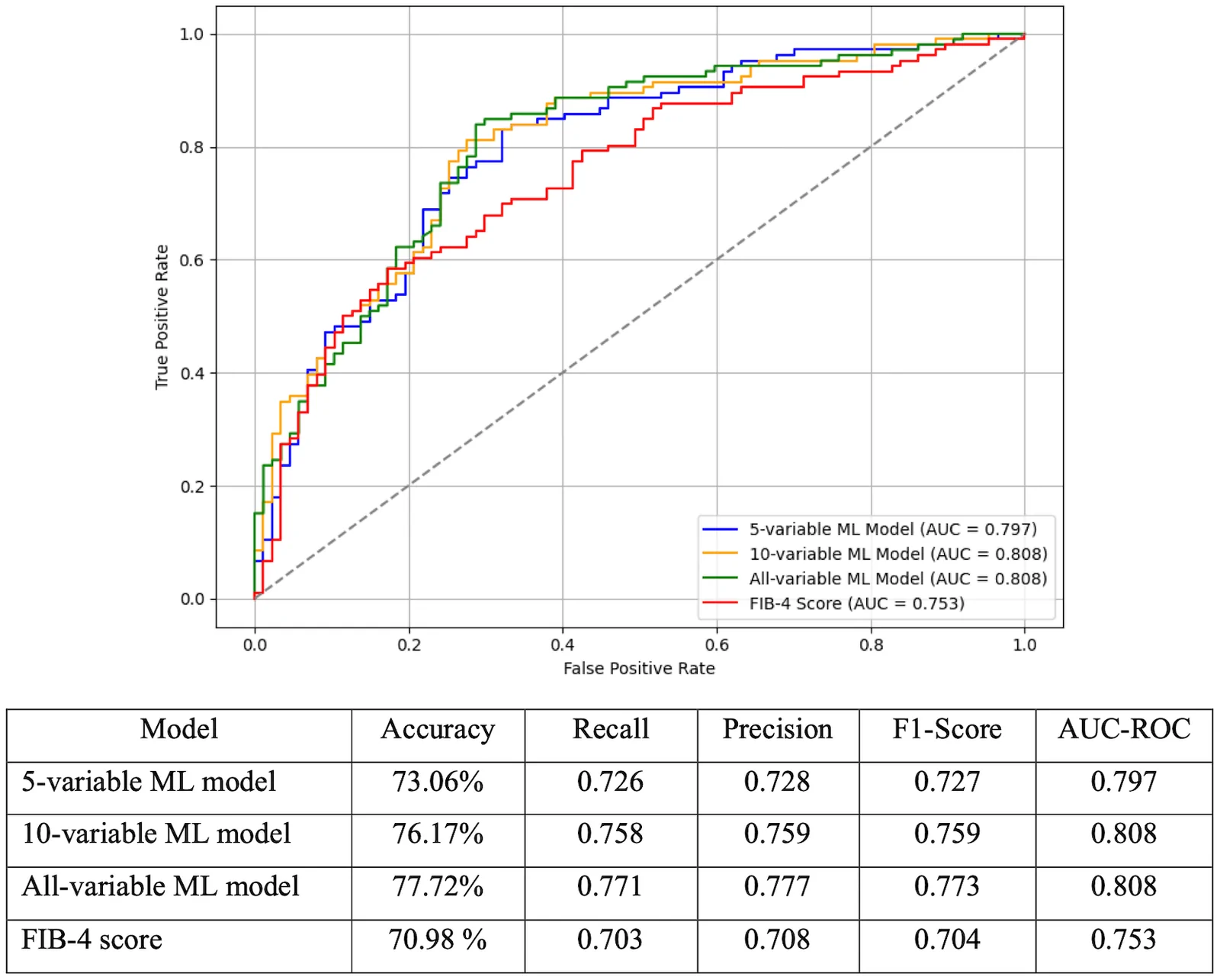
Comparison of predictive performances of the different ML models and the FIB-4 score fitted on the test sample. AUC-ROC: Area Under the Curve – Receiver Operating Curve, ML Machine learning.

In search of a parsimonious model suitable for low- and middle-income countries, we built models using the most important 5, the most important 10 and all variables separately. The performance comparisons of the models using 5, 10, and all variables are shown in Fig 2. The model using all the variables had the highest accuracy and precision, while the 10-variable model had comparable accuracy and precision but a slightly lower AUC-ROC. Therefore, all three ML models were validated in an external sample.

### External validation

The external validation sample consisted of 430 adults: 64.9% male, a mean age of 56.2 years, and 67.4% with SF. The baseline characteristics of the external validation cohort are shown in Table 3.

**Table 3:** Baseline characteristics of the external validation sample.

	Total N = 430
Age, mean(SD), years	56.2(10.9)
Age ≤ 65 years, N (%)	355 (82.6)
Age > 65 years, N (%)	75 (17.4)
Male, N (%)	277 (64.9)
Hypertension, N (%)	186 (43.3)
Dyslipidaemia, N (%)	199 (46.3)
Diabetes N (%)	220 (51.2)
Diabetic complications, N (%)	10 (2.3)
Hypothyroidism, N (%)	26 (6.1)
Family history of liver disease, N (%)	39 (9.1)
Current smokers, N (%)*	43 (13.9)
BMI, mean(SD) Kg/m2**	25.38 (4.4)
BMI ≥ 23 Kg/m2, N (%)	302 (71.1)

N number, SD standard deviation, BMI body mass index.

Missing cases: *20, **5.

**Fig 3:**
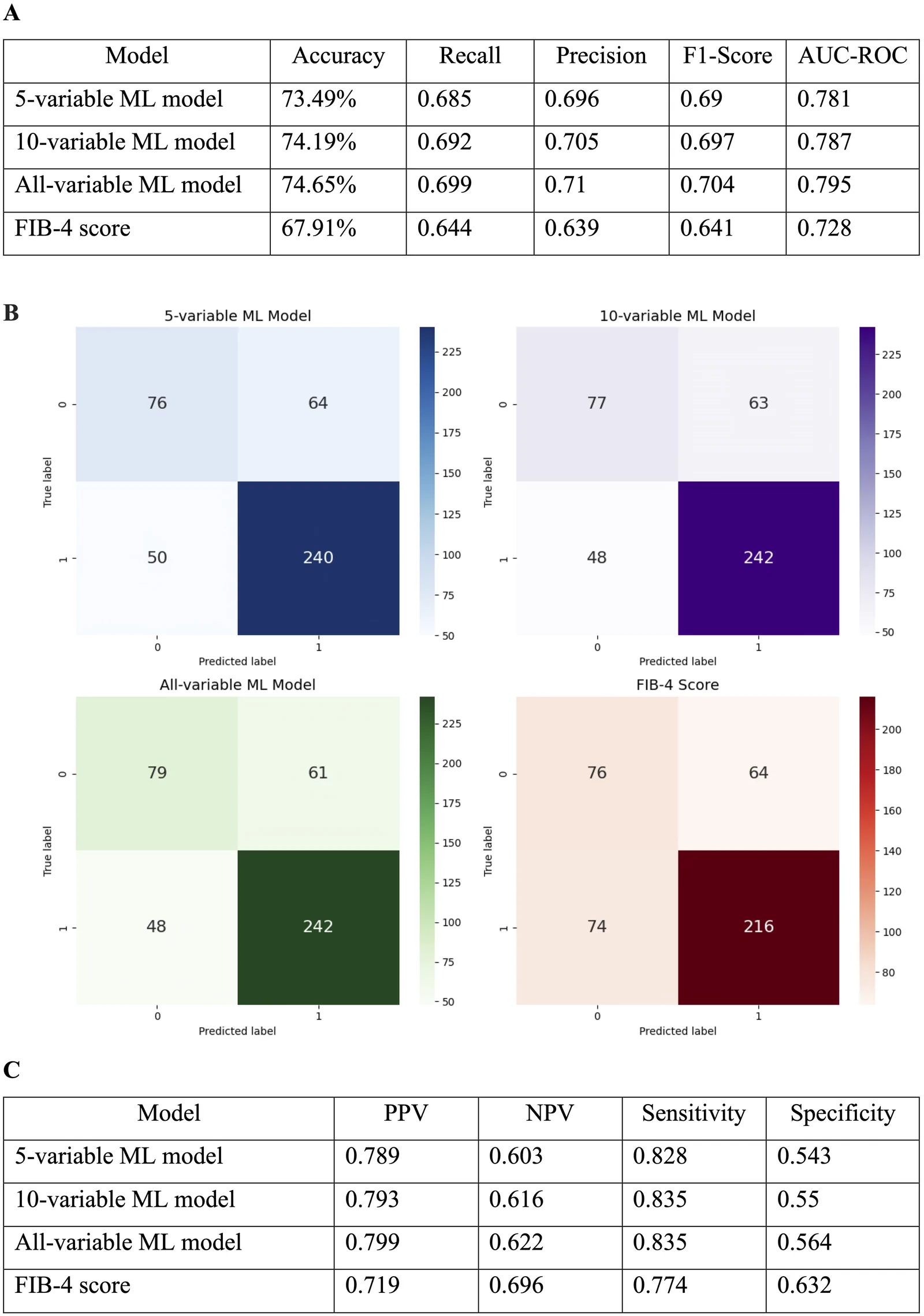
Comparison of the performances of ML models and the FIB-4 score as the screening tool in the MASLD screening algorithm. Panel A – Predictive performances, Panel B – Confusion matrices, Panel C – Diagnostic accuracy measures. AUC-ROC area under the curve, receiver operating characteristic curve, ML machine learning.

Comparisons of the performances of the 5-, 10-, and all-variable ML models and the FIB-4 score on the external validation sample are shown in Fig 3. All three ML models fitted on the external validation sample had better performances than the FIB-4 score in accuracy, recall, precision, and AUC-ROC (Fig 3, panel A). The corresponding AUC-ROCs of the 5-, 10- and all-variable ML models and the FIB-4 score were 0.781, 0.787, 0.795, and 0.728, respectively. The confusion matrices for the different ML models and the FIB-4 score used in the external validation sample to predict SF as the first step of the MASLD screening algorithm are shown in Fig 3, panel B. All the ML models had higher sensitivity and PPV than the FIB-4 score for predicting SF, with the all-variable ML model performing the best. The all-variable ML model had a PPV of 0.799 and an NPV of 0.622, compared with 0.719 and 0.696 for the FIB-4 score (Fig 3, panel C). The all-variable ML model correctly predicted fibrosis status in 321 (74.7%) patients, compared with 292 (67.9%) with the FIB-4 score. Using the all-variable ML model as the first screening step in the MASLD screening algorithm identified 26 (9.0%) more patients with SF while reducing 3 unnecessary VCTE referrals compared with using the FIB-4 score as the screening tool.

## Discussion

We observed that using ML-based models for screening in the first step of the MASLD screening algorithm, rather than the FIB-4 score, was more efficient at identifying SF in the South Asian samples studied. Using the all-variable ML model as the screening tool in the first step of the MASLD algorithm correctly identified fibrosis status in 74.7% of patients, with 9.0% more true-positive patients detected without unnecessary VCTE referrals compared with using the FIB-4 score as the screening tool. This finding is particularly valuable for resource-limited settings where VCTE is not freely available, as it allows optimal utilisation of limited resources by prioritising patients for VCTE, reducing unnecessary scans while increasing detection rates in cohorts. The 5-variable ML model even showed higher predictive performance than using the FIB-4 score, supporting the practical utility of ML models in clinical settings where data is limited.

Recent literature reports promising evidence for ML-based models outperforming well-known traditional risk prediction models [26,32–34,36,38]. Our findings add to the evidence, complemented by data from South Asians. Different ML models identified distinct parameters for predicting SF. Abdominal circumference, waist circumference, chest circumference, truncal fat, body mass index, ALT, GGT, platelet count, and age were among the most predictive variables identified by other studies, especially from Western countries, and we also identified those as predictive [27–31,34,35,38]. The Gut and Obesity in Asia (GO-ASIA) study found that age, AST, ALT, fasting plasma glucose, and platelet count were the top predictors of SF across eight Asian countries [35]. In addition, we identified several additional factors as being associated. We observed that, in this South Asian sample, AST, platelet count, BMI, diabetes mellitus, ALT, and age were the top 6 factors associated with SF, in descending order. In our sample, BMI had a stronger association with SF than age, unlike the FIB-4 score. We observed that using the four variables used in the FIB-4 score (AST, ALT, platelet count, and age) separately, instead of using the calculated FIB-4 score as variables in the ML models, was more predictive of SF, probably explaining the differential association of those factors in South Asians and white Caucasians. Further, we noted that the presence of diabetes mellitus was a significantly important risk factor in predicting SF in this South Asian sample, which is explained by the higher prevalence of diabetes in South Asians than in white Caucasians, while the association of SF and diabetes is already reported [39]. In addition, we identified that a family history of liver disease was a significant predictor of SF in this South Asian sample. The PNPLA3 gene has been identified as a robust genetic factor for increased liver fibrosis in MASLD and is observed in South Asians, predisposing those with MASLD to advanced fibrosis [40–43]. This may explain the association between family history of liver disease and SF we identified in this South Asian sample.

There are several strengths in our study. We analysed data of all consecutive patients with MASLD, eliminating selection bias. VCTE was performed by the same operator using the same fibro scanner, ensuring independence and avoiding operator-related bias. We studied more than 100 variables without prejudice to identify associations with SF, then selected the best predictive variables for SF. We calculated the sample size to achieve the required effect size. Medical officers prospectively interviewed patients before VCTE; therefore, data quality is good. We validated the model's predictions in an external cohort. However, some limitations of our study should be acknowledged. We studied only a Sri Lankan cohort, and therefore, findings may not be generalisable to all South Asians, but this study paves the way for the application of the same in other South Asian countries, as we identified important risk factors related to South Asians that were not identified as so important in white Caucasians.

## Conclusion

The new ML-based models, developed using non-invasive, freely available data, performed better than the FIB-4 score at detecting SF when used as the screening tool in the first step of the EASL MASLD screening algorithm in the studied cohorts of South Asians with MASLD. The all-variable ML model correctly identified fibrosis status in 74.7% of patients, with 9.0% more true-positive patients detected without unnecessary VCTE referrals compared with using the FIB-4 score as the screening tool.
